# Effects of a Circuit Training Program on Muscular and Cardiovascular Endurance and their Maintenance in Schoolchildren

**DOI:** 10.2478/hukin-2013-0036

**Published:** 2013-07-05

**Authors:** Daniel Mayorga-Vega, Jesús Viciana, Armando Cocca

**Affiliations:** 1 Department of Physical Education and Sport, University of Granada, Spain.; 2 Department of Physical Education and Sport, University of Granada, Spain.; 3 Department of Physical Education and Sport, University of Granada, Spain.

**Keywords:** physical fitness program, health-related physical fitness, muscular strength, cardiorespiratory fitness, school-based program, physical education

## Abstract

The purpose of this study was to evaluate the effects of a circuit training program along with a maintenance program on muscular and cardiovascular endurance in children in a physical education setting. Seventy two children 10–12 years old from four different classes were randomly grouped into either an experimental group (n = 35) or a control group (n = 37) (two classes for each group). After an eight-week development program carried out twice a week and a four-week detraining period, the experimental group performed a four-week maintenance program once a week. The program included one circuit of eight stations of 15/45 to 35/25 seconds of work/rest performed twice. Abdominal muscular endurance (sit-ups in 30 seconds test), upper-limbs muscular endurance (bent arm hang test), and cardiovascular endurance (20-m endurance shuttle run test) were measured at the beginning and at the end of the development program, and at the end of the maintenance program. After the development program, muscular and cardiovascular endurance increased significantly in the experimental group (p < 0.05). The gains obtained remained after the maintenance program. The respective values did not change in the control group (p > 0.05). The results showed that the circuit training program was effective to increase and maintain both muscular and cardiovascular endurance among schoolchildren. This could help physical education teachers design programs that permit students to maintain fit muscular and cardiovascular endurance levels.

## Introduction

Physical fitness is nowadays considered as one of the most important health markers in childhood ( Ortega et al., 2008 ). Consequently, in the last decades several countries have been promoting physical fitness improvement among young people in different ways ( Department of Health and Human Services, 1990 ). In many circumstances, schools have been considered the best setting in which children with low fitness levels can be identified and a healthy lifestyle can be promoted ( Ortega et al., 2008 ). Therefore, one of the main Spanish government strategies was focused on modifying school legislations in order to give health a more important role in the Educational System ( Ministerio de Educación y Ciencia, 2006 ). Schools are mainly attempting to increase the pupils’ health level by using measures such as the improvement of their physical fitness through physical education (PE) ( Ministerio de Educación y Ciencia, 2006 ). It has been concluded that the health promotion policies and physical activity programs should be designed to improve physical fitness, where strength and cardiovascular endurance are the most important health-related physical fitness components ( Ortega et al., 2008 ).

It is known that planning long-term fitness programs is the best way to improve these components ( Donnelly et al., 2009 ). Nonetheless, in the PE setting these programs cannot last the whole course or a large part of it since many curricular contents must be developed in a school year ( Ministerio de Educación y Ciencia, 2006 ). Consequently, in the PE setting we need to find short-term programs that could be also effective for the increment of fitness. One of the methodologies that meet these criteria could be the circuit training ( Dorgo et al., 2009 ; Granacher et al., 2011a ; Granacher et al., 2011b ). The circuit training effectively reduces the time devoted to training while allowing an adequate training volume to be achieved ( Alcaraz Ramón et al., 2008 ). Moreover, it permits a greater motor engagement time ( Lozano et al., 2009 ), which is a very important requirement for the success of a PE program. In addition, this methodology has multilevel effects on fitness, especially in beginners ( Alcaraz Ramón et al., 2008 ; Dorgo et al., 2009 ; Wong et al., 2008 ).

Other problem related to physical fitness is its expected decrease after a period of detraining. Several authors confirm that after 8 to 12 weeks of detraining children lose a significant part of the physical fitness gains obtained ( Da Fontoura et al., 2004 ; Faigenbaum et al., 1996 ; Ingle et al., 2006 ; Isaacs et al., 1994 ; Tsolakis et al., 2004 ). A possible solution for this problem could be the periodical introduction of short maintaining programs throughout the academic course. During these intervals, PE teachers would be able to develop other curricular contents and at the same time they could be improving the previous physical fitness gains. These programs could permit to keep the physical fitness level achieved without interfering in the normal course of the PE planning. Unfortunately, to our knowledge there are no studies addressing the effect of a physical fitness maintenance program in a PE setting. Consequently, the purpose of this study was to evaluate the effects of a circuit training program and its maintenance on muscular and cardiovascular endurance levels in children in a PE setting.

## Material and Methods

### Participants

Seventy two apparently healthy Spanish children (boys, n = 40; girls, n = 32) 10–12 years old (age 11.10 ± 0.38 years; body mass 43.29 ± 10.45 kg; body height 1.43 ± 0.07 m; body mass index 19.39 ± 3.90 kg/m ^2^ ) from four different PE classes of a public primary school participated in this study. For practical reasons and the nature of the present study (the intervention was focused on natural groups in a school context) a cluster randomized controlled trial was used. General profiles were assigned randomly to form one of the study groups (two classes for each group): experimental group (EG, n = 35), or control group (CG, n = 37). EG and CG consisted of two gender balanced groups (47% and 43% of girls, respectively).

All participants were urged to maintain their normal levels of physical activity outside of the supervised setting. Twenty-seven children in the EG (77%) and 27 children in the CG (73%) regularly participated (at least twice per week) in organized sports programs. Children and their legal guardians were fully informed about all the features of the study and were required to sign an informed consent form. The Ethical Committee of the University of Granada approved the study protocol.

### Measures

The participants were evaluated using the muscular and cardiovascular endurance tests included in the EUROFIT battery ( Council of Europe Committee for the Development of Sport, 1988 ), validated and standardized by the Council of Europe. The test sessions were carried out during the PE classes at the beginning and at the end of the development circuit training program (pretest and posttest), in order to see the changes that were produced. Subsequently, after a period of detraining and the application of the maintenance circuit training program, the participants were evaluated again (retest).

The tests were administered in an indoor sports center court with a non-slip floor, under the same environmental conditions, on the same day and at the same time for each student. A blind evaluation was carried out by two researchers following the standard protocol for each test. Each researcher assessed physical fitness with the same tests using identical equipment. Prior to the evaluation, the participants completed a standardized warm-up consisting of five minutes of running from low to moderate intensity. The order and a brief description of the test protocol are as follows:

*Sit-ups in 30 seconds test* (SUP). This test was used to measure abdominal muscular endurance. The participants laid supine on the mat with their knees flexed at an angle of 90 degrees and their feet flat on the floor, stabilized by a researcher. The participant’s fingers were to be interlocked behind their head. On the command ‘Go’, the participants’ elbows had to contact the knees and return to the starting position as many times as possible in 30 s. Each participant was allowed to perform the test once. The total number of sit-ups performed in 30 s was recorded.

*Bent arm hang test* (BAH). This test was used to measure upper-limbs muscular endurance. The participants had to maintain a bent arm position while hanging from a bar with hands in a pronated grip and at shoulder width. The participants’ chin had to be above the bar and held in this position as long as possible. The test ended when the participants’ eyes went below the bar. Each participant was allowed to perform the test once. The total time in seconds was retained.

*20-m endurance shuttle run test* (ESR). This test was used to assess cardiovascular endurance. All students ran between two parallel lines put 20 m apart, in the rhythm marked by a recorded beep. A researcher ran alongside the children to help them keep the desired pace. The starting speed was 8.5 km/h; and it increased 0.5 km/h every minute. The test ended when the child stopped running due to fatigue or failed to reach the line before the next signal for two consecutive times. Each participant was allowed to perform the test once. The last completed lap (timed in seconds) was recorded.

### Procedures

A circuit training program was applied to the EG during the PE classes under the supervision of a researcher. Firstly, the EG participants performed a development circuit training program twice a week on nonconsecutive days for eight weeks. They completed a total of 14 training sessions, since two classes coincided with festivals and could not be used. Then, after a period of detraining (four weeks) coinciding with Christmas holiday, the EG participants completed a maintenance circuit training program one session per week during four weeks. During the period of maintenance program, each session of maintenance was alternated with a normal class of PE according to the course planning designed by the teacher.

Each session lasted 50 minutes and consisted of a five-minute warm up during which children had to play a racing game, 40-minute circuit training, and two series of a 15–30 second cool-down of static stretching, primarily for the hamstrings and lumbar region ( Table 1 ). All exercises were fully explained and previously demonstrated by the researcher, and children were asked to try them during a few minutes before starting the first session of the intervention. According to previous studies carried out in the PE setting ( Dorgo et al., 2009 ; Granacher et al., 2011a ; Granacher et al., 2011b ), the intervention was organized in a circuit program. One circuit of eight stations was developed, and then repeated twice in each session. Each station consisted of an exercise lasting from 15 to 35 seconds (extended progressively from the first session to the last), and the rest time between them was of 45-25 seconds (gradually reduced during the program). The increase of the work time and the decrease of the rest time along the intervention were based on the training load progression principle.

During the work time the students should complete as many repetitions as possible in a controlled manner. As other studies show, the last repetition of each set represents the momentary muscular fatigue ( Faigenbaum et al., 2002 ; Faigenbaum et al., 2005 ). In order to achieve it, the children were offered three levels of difficulty in each station ( Table 1 ), so that the intensity of exercise was best suited to each student. All participants began at the first level of difficulty, and when a student could perform more than one repetition per second, he/she was allowed to advance to the next level. With the aim of developing cardiovascular endurance, at the end of each circuit all students simultaneously executed an additional stage consisting of a five-minute endurance racing game. The researcher gave positive feedback to motivate participants in achieving it ( Badami et al., 2011 ).

During the development and maintenance programs the EG executed the circuit training,while the CG participated in traditional games, basketball and volleyball activities. However, during the maintenance program the EG alternated one session of physical fitness maintenance with other activities such as basketball and volleyball. No participant was allowed to carry out any physical fitness training outside of the supervised setting.

### Analysis

Descriptive statistics (means and standard deviations) for age, body height, body mass index, and muscular and cardiovascular endurance results were calculated. The Student’s t test for independent samples was used to study the differences of the general characteristics between groups. As the BAH variable did not follow a normal distribution, the data was transformed using a logarithm ( Bland and Altman, 1996 ). Because a higher precision was required for ESR test performance, the final time spent in the test was expressed in seconds, instead of stages or half stages, and it was used for statistical analysis ( Ruiz et al., 2011 ). A two-way analysis of variance (ANOVA) was applied over the dependent variables (SUP, BAH, ESR) using groups (EG, CG) and time factors (pretest, posttest, retest). For the post-hoc analyses, α values were corrected using the Bonferroni adjustment. The Hedges’ g effect size was used to determine the magnitude of treatment effects ( Hedges, 2007 ). The test-retest reliability for muscular and cardiovascular endurance tests was estimated using the intraclass correlation coefficient from two-way ANOVA (ICC3,k) ( Shrout and Fleiss, 1979 ). Furthermore, 95% interval of confidence was calculated. All statistical analyses were performed using the SPSS version 15.0 for Windows (SPSS® Inc., Chicago, IL). The statistical significance level was set at p < 0.05.

## Results

All students completed the development training program and 67 the maintenance training program according to previously established norms (no more than two classes were missed in the development training program, and none were missed in the maintenance training). Retest data of four participants from the EG and one from the CG were excluded due to missed classes in the maintenance training program and absence in the retest session test, respectively. The EG participants finally considered for analysis obtained an average attendance of 94% and 100% in the development and maintenance training program, respectively. The Student’s t for independent samples results did not show statistically significant differences in the general characteristics between EG and CG.

*Sit-ups in 30 seconds test* . The EG had significantly greater gains in SUP compared to the CG [F(2, 63) = 4.636; p = 0.011; η ^2^_ p_ = 0.069; P = 0.773] ( Table 2 ). The ANOVA with Bonferroni adjustment showed that the EG increased significantly from pretest to posttest (p = 0.026) and from pretest to retest (p = 0.004). Nevertheless, the difference from the posttest to the retest for the EG was not statistically significant (p = 0.105). No significant differences were found for the CG (p = 1.000). The test-retest reliability for the SUP was 0.86 (0.73–0.93).

*Bent arm hang test* . Significantly greater gains were found for the EG compared to CG [F(2, 63) = 5.994; p = 0.003; η ^2^_ p_ = 0.087; P = 0.875]. The EG participants significantly increased FAH from pretest to posttest (p = 0.009) and from pretest to retest (p < 0.001). For the EG, the improvement from the posttest to the retest approached statistical significance (p = 0.065). No differences were found for the CG (p ≥ 0.324). The test-retest reliability for the BAH was 0.95 (0.90–0.97).

*20-m endurance shuttle run test* . The EG had significantly greater gains in ESR compared to the CG [F(2, 64) = 5.230; p = 0.007; η ^2^_ p_ = 0.076; P = 0.824]. The ANOVA with Bonferroni adjustment showed that the EG increased significantly from pretest to posttest (p = 0.015) though improvement from the pretest to the retest approached statistical significance (p = 0.088). No significant differences were found from posttest to retest for the EG (p = 0.210). No differences were found for CG (p ≥ 0.975). The test-retest reliability for the ESR was 0.90 (0.81–0.95).

## Discussion

The results of the present study show that it is possible to develop both muscular and cardiovascular endurance by means of an eight-week circuit training program in the PE setting. Previous studies in which children performed an extra-curricular circuit training program confirmed a significant improvement on both muscular and cardiorespiratory fitness ( Annesi et al., 2005 ; Ignico and Mahon, 1995 ; Wong et al., 2008 ).

Previous studies in which children performed an extra-curricular circuit training program confirmed a significant improvement on both muscular and cardiorespiratory fitness ( Annesi et al., 2005 ; Ignico and Mahon, 1995 ; Wong et al., 2008 ). Nevertheless, the design and the procedure of the present study depended on many aspects related to the school context as previously discussed in this manuscript. Likewise, due to the lack of special machines in a PE setting, in the present study body weight, elastic band and ball exercises ( Annesi et al., 2005 ; Faigenbaum and Mediate, 2008 ; Flanagan et al., 2002 ) were used instead of specific strength equipment ( Granacher et al., 2011a ; Granacher et al., 2011b ).

One of the main objectives of the PE teachers at these educational levels is to make the pupils active as long as possible during the classes. With the circuits method the pupils can easily reach the minimum motor engagement time ( Lozano et al., 2009 ) at the same time they execute many types of exercises. This is the best way to make the most of the time at a PE teacher’s disposal, especially when classes are few and short-lasting and there are many contents to develop ( Ministerio de Educación y Ciencia, 2006 ). Thus, the present results indicate that the design proposed in this research could be effective for PE classes. In this line, Dorgo et al. (2009) carried out a circuit training program with adolescents in the PE setting. These authors found a statistically significant improvement for both muscular strength and cardiovascular endurance when the circuit training was complemented with endurance training.

One of the most important outcomes of this study was that a maintenance program carried out once a week in four weeks could be effective to maintain the gains previously obtained. As explained before, the majority of studies coincide in eight weeks setting as the period of inactivity determining the complete loss of previous physical fitness gains ( Faigenbaum et al., 1996 ; Isaacs et al., 1994 ; Tsolakis et al., 2004 ).

In the present study the sum of the periods of detraining and maintenance was eight weeks, thus an unsatisfactory design of the maintenance program should have matched a decrease (or the complete dissipation) of the physical fitness profits. Nevertheless, results were positive since the muscular and cardiovascular endurance was maintained after these weeks.

In line with the present study, DeRenne et al. (1996) found out that a maintenance program carried out once a week in pubescent basketball players was efficient to retain strength. However, Blimkie et al. (1989) found that a maintenance program carried out once a week in pre-pubescent children was not efficient to retain strength. Unfortunately, previous studies that examined the maintenance of cardiovascular endurance in youth were not found. In addition, the previous studies were carried out in an extra-curricular period and not in a PE setting. Furthermore, the researchers applied the maintenance program just after the training program, that is, without a period of inactivity between development and maintenance. In the present study, a maintenance program was applied after a period of detraining because it is the most common situation in normal PE planning (due to the typical alternation of holidays, academic periods and the need to teach other curricular contents in the PE classes). Consequently, the design of the present study seems to be suitable for the school environment as it respects all the features and norms established in it. Moreover, it should be effective for increasing the strength and cardiovascular endurance values and then maintaining them during larger periods.

In conclusion, the present study suggests that it is possible to develop and maintain muscular and cardiovascular endurance through a short-term program in the PE setting. Maintenance programs appear to be necessary in the school context to make the physical fitness training effective and feasible within a school plan, permitting at the same time the regular development of other curricular contents. Their utilization could permit the PE teacher to design programs that guarantee the maintenance of previous muscular and cardiovascular endurance gains in a few sessions. Even though more research is needed to confirm these results, the maintenance program could become a principal element to normal PE planning in the future. A limitation of the present study was the fact that the intervention program did not include many playful tasks. At these ages in PE classes it is important to develop contents mainly through ludic activities. Future interventions should focus on physical fitness programs based on stations with games, as well as the effect of the combination of different frequencies and durations of maintenance training.

## Figures and Tables

**Table 1:** *
Circuit training session
*

Phase (time)/ Exercises	Intensive progression (level 1/ 2/ 3) ^a^	Material
Warm-up (5 min)		
Racing games		
Main part (40 min)		
Circuit training stations		
a. Throwing from chest	1kg/ 1.5kg/ 2kg	MB
b. Rowing	Low/ medium/ high resistance	Elastic band
c. Going up-down	Body weight/ +1kg/ +2kg	Swedish bench, MB
d. Triceps extension	Low/ medium/ high resistance	Elastic band
e. Biceps curl	Low/ medium/ high resistance	Elastic band
f. Skipping rope	Micropause/ with/ without rebound	Rope
g. Crunches	Arms stretched forward/ chest/ backward	Mat
h. Bridging	Body weight/ +1kg/ +2kg	Mat, MB
Additional station		
i. Racing games		
Cold-down (5 min)		
Static stretching		

*MB = Medicine ball;*

a *All participants began at the first level of difficulty.*

*When a student could perform more than one repetition per second was allowed to advance to next level.*

**Table 2:** *
Muscular and cardiovascular endurance performance for the development and maintenance circuit training program
*

Group	Pretest (1) (M ± SD)	Posttest (2) (M ± SD)	Retest (2) (M ± SD)	p	Effect size		
					1–2	2–3	1–3
SUP (nº)							
Experimental	20.37 ± 4.21	22.09 ± 3.70 *	23.10 ± 3.88 ^††^	<0.05	0.44	0.22	0.68
Control group	17.95 ± 4.94	17.64 ± 5.89	17.57 ± 5.76				
BAHa (s)							
Experimental	11.63 ± 9.93	14.65 ± 12.75 **	20.80 ± 19.07 ^†††^	<0.01	0.38	0.31	0.65
Control group	16.87 ± 18.84	14.13 ± 14.09	16.11 ± 15.95				
ESR (s)							
Experimental	160.71 ± 94.33	198.71 ± 98.43 *	186.00 ± 84.76	<0.01	0.48	−0.11	0.37
Control group	191.78 ± 96.66	184.06 ± 96.12	181.78 ± 100.22				

*M = Mean; SD = Standard deviation; SUP = Sit-ups in 30 seconds test;*

*BAH = Bent arm hang test; ESR = 20-m endurance shuttle run test; BAH ^a^ = for statistical analysis the raw data were transformed by the logarithm; p = significance level from two-way analysis of variance; Effect size = Hedges’* g *effect size.*

Post-hoc analyses with Bonferroni adjustment: Change statistically significant from pretest to posttest (*p < 0.05, **p < 0.01);

*Change statistically significant from pretest to retest (††p < 0.01, †††p < 0.001)*
